# Identification of Superior Alleles for Seedling Stage Salt Tolerance in the USDA Rice Mini-Core Collection

**DOI:** 10.3390/plants8110472

**Published:** 2019-11-05

**Authors:** Jai S. Rohila, Jeremy D. Edwards, Gioi D. Tran, Aaron K. Jackson, Anna M. McClung

**Affiliations:** 1 Dale Bumpers National Rice Research Center, United States Department of Agriculture (USDA)—Agricultural Research Service (ARS), Stuttgart, AR 72160, USA; jeremy.edwards@usda.gov (J.D.E.); aaron.jackson@usda.gov (A.K.J.); anna.mcclung@usda.gov (A.M.M.); 2 Genetics and Plant Breeding Division, Cuu Long Delta Rice Research Institute, Tan Thanh, Thoi Lai, Can Tho 905660, Vietnam; tdgioi@gmail.com

**Keywords:** rice, salt stress, GWAS, salt tolerance, SNP, abiotic stress

## Abstract

Salt stress is a major constraint to rice acreage and production worldwide. The purpose of this study was to evaluate the natural genetic variation available in the United States Department of Agriculture (USDA) rice mini-core collection (URMC) for early vigor traits under salt stress and identify quantitative trait loci (QTLs) for seedling-stage salt tolerance via a genome-wide association study (GWAS). Using a hydroponic system, the seedlings of 162 accessions were subjected to electrical conductivity (EC) 6.0 dS m^−1^ salt stress at the three-to-four leaf stage. After completion of the study, 59.4% of the accessions were identified as sensitive, 23.9% were identified as moderately tolerant, and 16.7% were identified as highly tolerant. Pokkali was the most tolerant variety, while Nerica-6 was the most sensitive. Adapting standard International Rice Research Institute (IRRI) protocols, eight variables associated with salt tolerance were determined. The GWAS of the URMC, using over three million single-nucleotide polymorphisms (SNPs), identified nine genomic regions associated with salt tolerance that were mapped to five different chromosomes. Of these, none were in the known Saltol QTL region, suggesting different probable genes and mechanisms responsible for salt tolerance in the URMC. The study uncovered genetic loci that explained a large portion of the variation in salt tolerance at the seedling stage. Fourteen highly salt-tolerant accessions, six novel loci, and 16 candidate genes in their vicinity were identified that may be useful in breeding for salt stress tolerance. Identified QTLs can be targeted for fine mapping, candidate gene verification, and marker-assisted breeding in future studies.

## 1. Introduction

Rice is a staple food for billions of people, and it is the only food crop that provides more than 50% of daily calories by direct consumption to more than half of the world’s population [1]. According to recent projections, the worldwide demand for rice is expected to increase [2], but the long-term environmental sustainability of rice production is under question due to its large fresh-water irrigation requirements. Rice is generally grown in flooded paddies and surface water is often supplemented with water pumped from underground wells, which sometimes is of poor quality due to the presence of high amounts of dissolved salts, especially in depleted aquifers [3,4]. Moreover, a rice crop can be exposed to salt stress due to natural soil salinity or by salt-water intrusion in irrigation resources and fields in low-lying coastal areas. Salt-water intrusion in rice fields has occurred due to sea-level rise and inundations during tidal surges from tropical storms or tsunamis along the Gulf Coast in the USA, Japan, Vietnam, and elsewhere [5,6]. Rice is susceptible to the quantity and the quality of irrigation water, as it directly affects rice yield and grain quality [7]. Salt stress is one of the most important yield-limiting abiotic stresses in flooded lowland rice production areas and because there are limited opportunities for the mitigation of salt-water intrusion, efforts toward crop adaptation through breeding are needed. In general, rice is affected by saline conditions above 2 dS m^−1^ at the seedling stage and at reproductive stages [8,9,10]. Salinity conditions in the field during early plant growth stages affect stand establishment, and later in the growth cycle can influence many morphological (e.g., root length and diameter, photosynthetically active canopy area, plant height), physiological (e.g., net photosynthesis, stomatal conductance), and biochemical processes (e.g., accumulation of reactive oxygen species, osmolytes, ion homeostasis), which eventually contribute to grain yield reduction [9,11]. 

Multiple studies have identified several basic molecular mechanisms by which plants respond to and confer resistance to salinity stress [12,13,14,15]. These include: transporters, detoxification of Na^+^-induced reactive oxygen species, storage (or compartmentalization) of salts in the apoplast and vacuole, secretion of salt directly out of the plant system via salt glands, an increase of osmolytes, and many more [16,17,18]. Knowledge of the genes responsible for these various mechanisms is critical for providing effective molecular tools for rice-breeding programs aimed at improving various aspects of salt tolerance.

Molecular markers have been identified that are linked to easily measurable phenotypic traits that convey salt tolerance such as shoot and root dry weight, plant height, root length, and Na+/K+ accumulation/exclusion in the shoot [19]. Research on the genetics of salt tolerance, including identification of the Saltol quantitative trait loci (QTL) [20], has aided in the adaptation of rice plants to salt stress, and has been found to primarily control shoot Na^+^/K^+^ homeostasis. However, considering the numerous processes that salt stress impacts in plants, combining multiple QTLs is needed to achieve higher levels of tolerance throughout the plant growth cycle [19]. Prior to the rice genome being sequenced, salt–stress tolerance mutants and major genes were mapped with the use of low-throughput markers such as: RFLP, RAPD, AFLP and SSR [20,21]. Genome sequencing has enabled the high-resolution mapping of quantitative traits using large high-throughput panels of single-nucleotide polymorphisms (SNPs) [22,23,24,25]. These molecular markers coupled with marker-assisted selection (MAS)-based breeding programs offer a means of incorporating salt tolerance into recently developed high-yielding germplasm [26].

However, the continued development of salt tolerant lines through breeding will require the identification of germplasm possessing genetic variability across a wide spectrum of salt tolerance-related traits. Compared to biparental mapping populations, diversity panels provide a much larger pool of genetic resources for the discovery of beneficial genetic variants (or alleles) from landraces and germplasm that can be examined for traits that are important components of salt stress tolerance and their associated QTLs [19,27]. The dissection of these traits by phenotyping and genotyping will reveal new insights into the biological mechanisms underlying these phenotypes. Numerous diversity panels have been developed and extensively genotyped using various platforms [28,29,30,31,32,33,34]. The United States Department of Agriculture (USDA) rice mini-core collection (URMC) is a subset of 217 accessions containing similar phenotypic, genotypic, and geographical diversity as present in the USDA rice core collection of 1794 accessions representing rice-growing countries around the globe [35,36]. The URMC has been successfully used by rice researchers for identifying sources of germplasm and novel QTLs for several important traits such as: grain yield [35,37,38], grain yield components and harvest index [39], silica content in hulls [40], grain quality [23], grain protein content [41], grain starch quality [42], sheath blight resistance [43,44], and cold tolerance at seed germination and the seedling stage [45]. These evaluations have provided insights into the beneficial phenotypic and genotypic diversity available in the URMC for rice breeding programs.

The purpose of this study was to identify novel sources of tolerance to salt stress at the seedling stage across the diversity of *Oryza sativa* represented by the URMC, to compare the range of phenotypic diversity for salt tolerance in the URMC to that of current international salt tolerant varieties, and to identify salt tolerance-associated genomic regions to enable the introduction of superior salt tolerance alleles into new rice varieties. 

## 2. Results 

### 2.1. Phenotypic Variation for Seedling Salt Tolerance Traits in USDA Rice Mini-Core 

We evaluated a panel of 162 diverse rice (*Oryza sativa* L.) accessions from URMC that represented four rice subpopulations: *Tropical japonica* (TRJ), *Temperate japonica* (TEJ), *Indica* (IND), and *Aus* (AUS) [35]. Six cultivars were included as salt-sensitive (IR29, Nerica 6, A69-1) and salt-tolerant (Pokkali, IR45427-2B-2-2B-1-1, IR65196-3B-5-2-2) checks [17,46,47]. A detailed view of all the traits measured in this study is provided in Table 1a,b. This study successfully differentiated salt-tolerant genotypes from salt-sensitive genotypes as evident by the Least Squares Means (LSMeans) (Table 1a) and the range values (Table 1b) of salt stress injury (SSI) scores. The day 16 (d16) LSMeans and range values in the sensitive checks were 7.53 and 5.2–9.00, respectively compared to 2.32 and 1.00–4.60 for the tolerant checks. The accessions in URMC showed a wide variation for SSI scores on d10 and d16, which ranged from a value of 1 to 9 with an LSMean of 6.39 on d16. Among the four subpopulations in the URMC, TRJ was identified as the most sensitive, with a mean SSI score of 5.63 on d10, and 7.55 on d16. The IND subpopulation (and two accessions in the TEJ) was the most tolerant. On average, the sensitive checks only increased in plant height by 8.25 cm following 10 d of salt exposure, whereas the tolerant checks grew 22.44 cm (Table 1a). The URMC and breeding lines from Vietnam were intermediate to the checks in plant height (PHT), and there were no significant differences among the four subpopulations at either d10 or d16. A similar trend was observed for the change in number of green leaves following 14 d of salt stress, with the URMC and breeding lines from Vietnam being intermediate to the checks. The subpopulations overlapped in number of green leaves, but the TRJ group was more senescent than the IND. A similar trend was observed for total, shoot, and root biomass. For the other traits that were measured, subpopulations with low SSI scores were associated with higher values for biomass, Δ green leaf number, and, although not significant, Δ PHT (Table 1a). As shown in Table 1b, a wide range in variation was observed for all these traits in URMC and among the lines from Vietnam; however, except for the SSI d10 score, there were no accessions as tolerant as the tolerant checks. 

**Figure 1:**
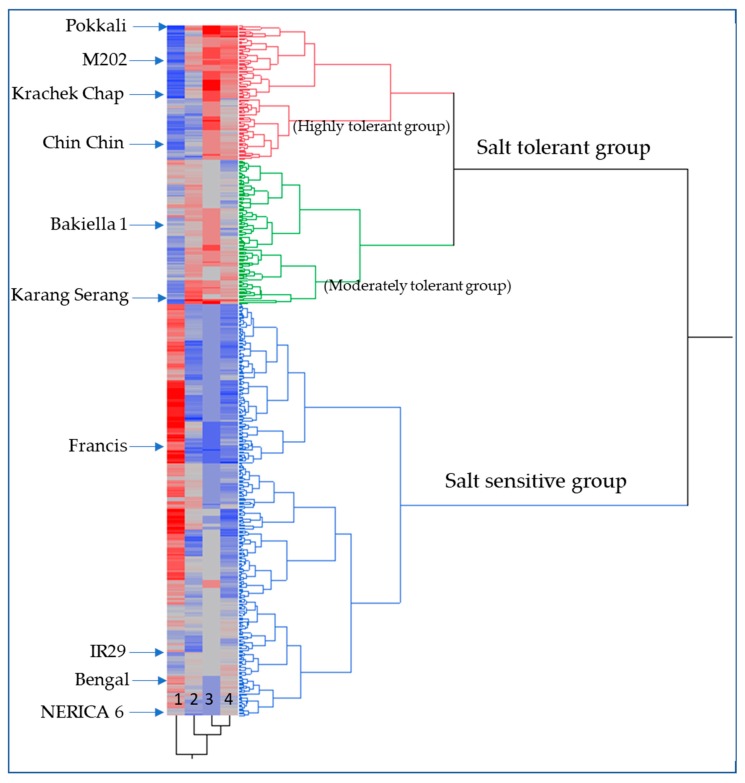
Two-way phenotypic dendrogram showing three clusters for variation in salt tolerance among 162 rice accessions including 123 URMC accessions. Four early vigor traits associated with salt stress tolerance at the seedling stage were used for constructing the dendrogram and are denoted by numbers: 1: SSI score d16; 2: Δ PHT d16; 3: Δ green leaf#; and 4: Total biomass plant^−1^ on the x-axis of the heatmap. The blue color illustrates low values, while the red color illustrates high values for the measured trait.

A two-way dendrogram was constructed based on the SSI score at d16, ΔPHT, Δ green leaf number, and total biomass plant^−1^ (Figure 1). The results demonstrate clear differences between the tolerant (e.g., Pokkali) and the sensitive check cultivars (e.g., IR 29). Although the majority of URMC accessions were clustered as susceptible, there were a number of accessions that were deemed highly or moderately tolerant. 

For a better understanding of the relationships among the measured traits in these 162 accessions, we computed Pearson’s correlation coefficient between each trait combination (Table 2). All traits were highly correlated with each other and contribute to the growth and senescence factors that influence the SSI scores. The Δ green leaf number had a strong negative correlation with SSI scores at both d10 (*r* = −0.80, *p* < 0.0001) and d16 (*r* = −0.86, *p* < 0.0001) and a strong positive correlation with the other measured traits such as shoot biomass (*r* = 0.71, *p* < 0.0001) and root biomass (*r* = 0.72, *p* < 0.0001). 

### 2.2. Identification of SNPs and QTLs Associated with Early Vigor and Tolerance for Salt Injury under Salt Stress

**Figure 2:**
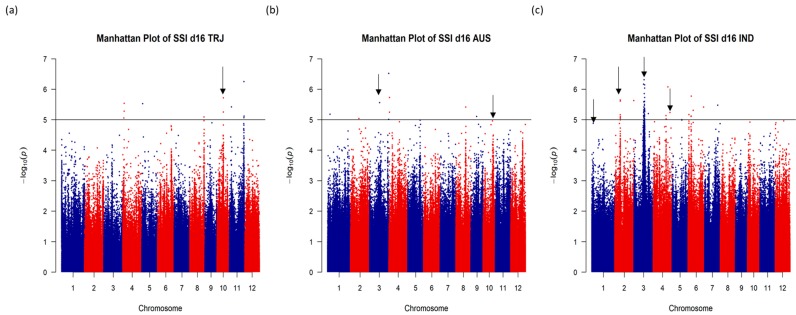
Genome-wide association analysis for salt stress injury score (SSI) on day 16 of salt stress treatment using single-nucleotide polymorphisms (SNPs) from the USDA rice mini-core. Manhattan plots are shown for three rice subpopulations: (**a**) *Tropical japonica* (TRJ), (**b**) *Aus* (AUS), and (**c**) *Indica* (IND). The SNP positions are shown across the 12 rice chromosomes (X axis) with a −log10 (p) value for each SNP (Y axis). The horizontal black line at −log10(p) = 5 is the significance threshold used in the analysis. Black arrows indicate approximate regions for the representative significant SNPs.

To identify genomic regions that were associated with salt tolerance in rice, we performed a genome-wide association study (GWAS) using 3.2 million SNP markers available for the URMC and mapped eight traits that were highly correlated with salt tolerance at the seedling stage. Manhattan plots of GWAS results and the identified segments were investigated, and nine genomic regions of interest were selected based on the significance of association across the traits, number of significant SNPs, and allele frequencies. The nine selected genomic regions were represented by nine unique SNPs, which were distributed on five different chromosomes of rice and were associated with one or more of the eight studied traits. Based on proximity to the centromere and lack of recombination, two selected regions on chromosome 3 were merged into a single region leaving a total of eight selected regions, seven of which were significantly associated with the SSI score and one that was associated with plant height. Figure 2 shows the approximate locations of the seven SSI d16 score-associated representative SNPs (Table 3a,b). Manhattan plots and respective quantile–quantile (Q-Q) distributions are presented as Figure S1. Table 3a shows the selected SNP chromosome positions (in base pairs, bp) with reference and alternate alleles, frequencies for the reference allele in different subpopulations (based on 3000 sequenced rice accessions [48,49]), and the subpopulations with significant SNP–phenotype associations. Additionally, information on all the significant regions was identified, and overlapping significant regions across all subpopulations and all traits are provided in Tables S1 and S2. 

The effects of each of the selected alternate alleles (relative to the reference allele) were calculated using the associated phenotypic traits in the subpopulation(s) where the significant association was detected (Table 3b). Each of the selected SNPs, except for SNP 1_33398135, were found to be associated with SSI score, Δ green leaf number, and root and shoot biomass. Three SNPs were significantly associated with Δ PHT, SSI score, Δ green leaf number, and root and shoot biomass. SNP 1_33398135 was associated with only one measured trait, Δ PHT. The highest observed allele effect for Δ PHT was 3.998 for SNP 1_33398135. The total phenotypic variance (Rsquare) of the associated trait explained by the identified selected SNP ranged from 6% to 71% with a median value of 33%. 

**Figure 3:**
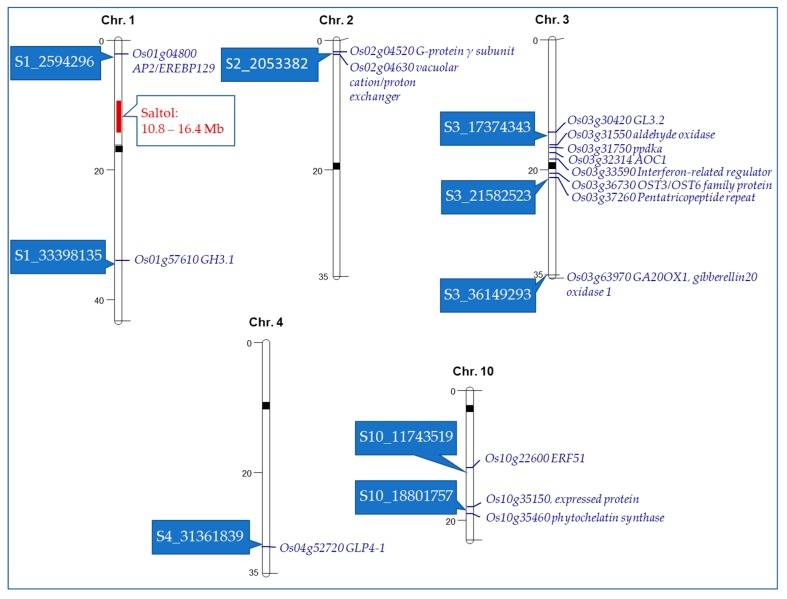
Physical location of candidate genes on five rice chromosomes based on identified SNPs associated with various salt tolerant traits at the seedling stage. Saltol, a major quantitative trait loci (QTL) [20], is shown for reference. SNPs are shown in rectangular boxes on the left side of the chromosomes. The candidate genes are shown in italics on the right side of the chromosomes.

To understand the potential of these SNPs in providing salt tolerance, putative candidate genes within the eight selected chromosomal regions were identified. In each chromosomal region, the SNP with lowest p-value was considered as the peak SNP. For identifying putative genes, we took an approach of selecting the genes close to the peak SNP and considering only those that were annotated by the rice scientific community (Oryzabase, MSU, RAP-DB), and published reports were used as evidence of the possible roles of the identified SNPs. Our analysis resulted in 16 candidate genes (Table 4), which are physically shown on each chromosome in Figure 3. A number of genes shown in this table have been found to encode transcription factors, including functional proteins such as Apetala2 (AP2) and Oligosaccharyltransferase3 (OST3), respectively. Additionally, there were several genes identified in this study that have not been implicated as having a direct role in salt tolerance, but are shown to have broader roles in overall abiotic stress tolerance. These genes may serve as novel targets in improving our understanding of salt tolerance in rice at the seedling stage. 

## 3. Discussion 

Here, we present the results from a study with 162 rice accessions coming from the URMC diversity panel (*N* = 123) that included four subpopulations of *Oryza sativa* L., along with 24 entries from a Vietnam breeding program, seven entries from US breeding germplasm, and six varieties that have been documented to display a range in susceptibility for salt exposure. Previously, it had been reported that seedling vigor under salt stress is an effective method of selection in rice breeding programs [63]. Thus, we grew the accessions hydroponically and, at the 3–4 leaf stage, rice seedlings were exposed to a constant EC 6.0 dS m^−1^ salt stress for 16 days. Genomic and phenotypic data were analyzed using 118 of the accessions that had been resequenced [24] to identify salt tolerant genotypes, QTLs, and SNPs using GWAS. 

We demonstrated that four traits (SSI score, ΔPHT, Δ green leaf number, and total biomass plant^−1^) were effective at differentiating salt-tolerant from salt-sensitive genotypes, and we observed significant variation in the URMC for the tolerance traits (Table 1). While increased plant height, shoot biomass, and root biomass are important for salt tolerance at the seedling stage, the ability to produce new leaves and maintain the functions of pre-existing leaves is also important for rice seedlings to remain physiologically and biochemically sustained under salt stress. Although none of the accessions were more tolerant than Pokkali, a known salt-tolerant genotype, 14 URMC accessions were statistically clustered as highly tolerant (Figure 1). In addition, about half of the advanced lines from Vietnam’s salt tolerance breeding program were also scored as highly tolerant (data not shown). Due to the concern of salt-water intrusion in many lowland rice-growing areas in Vietnam, they have effectively deployed the Saltol allele from Pokkali in a number of these varieties. However, there were several accessions in the URMC that performed better than the advanced salt-tolerant Vietnamese varieties but lacked the Saltol allele, indicating that there is the opportunity to pyramid diverse alleles to make additional gains in salt tolerance. Moreover, gains that come from multiple genes may act through synergistic mechanisms beyond what might be predicted from the estimated additive effects of each allele alone, and that is where trait-based modeling for salt tolerance in rice could support rice-breeding programs [64].

SSI scores, taken at 10 and 16 d post exposure to salt, were highly correlated (*r* = 0.90, *p* < 0.0001) and, as expected, the plants received a lower SSI score (less senescence) at the d10 rating (Table 1). This indicates that evaluations of germplasm for salt tolerance can be effectively determined at this earlier time point, which will increase the speed of selection. A strong correlation was determined between Δ green leaf number and root biomass (*r* = 0.7286, *p* < 0.0001). This suggests that maintaining a photosynthetically active leaf area under salt stress conditions is an adaptive tolerance mechanism [65]. Further, a stable or increased number of green leaves and biomass in tolerant germplasm under salt conditions may allow more salt to be sequestered in the vacuoles of roots and shoots, as excessive concentrations of Na^+^ in cytoplasm is detrimental to the physiological and biochemical processes of rice plants. The vacuolar sequestration of Na^+^ is known to support better seedling survival and plant growth under such stressed conditions [66,67]. 

Lee et al. [68] reported that tolerance to salinity was greater in *Indica* than *Japonica* subspecies. Our GWAS analysis was conducted with 118 URMC accessions that were represented by about twice as many *Indica* accessions than either the *Tropical japonica* or *Aus* subpopulations, affording the opportunity of identifying novel salt-tolerant alleles from *Indica* that would benefit US breeding programs, which are predominantly TRJ-based. GWAS analysis utilizing over three million SNPs identified nine unique SNPs that were highly associated with eight desirable salt tolerant traits. 

In GWAS, the ability to detect a phenotypic association with a SNP is dependent on the allele frequency of the SNP and linkage disequilibrium with the functional variant. There is low statistical power to detect associations with rare alleles, and SNPs with low minor allele frequency are filtered out in GWAS pipelines. When SNP–phenotype associations were found in only some subpopulations, this was often a consequence of little or no SNP variation in the subpopulation(s) lacking the association (Figure S2). For some SNPs, there were moderate allele frequencies present in some subpopulations where associations were not detected, and other subpopulations with significant associations. This may suggest that the SNP was not in linkage disequilibrium with the functional variant in all the subpopulations or that the genetic background of the subpopulation may be influencing the genetic pathways involved in salt tolerance. 

Considering the complex nature of salt stress tolerance, each identified SNP may have a small individual effect; thus, considering the aggregate effects of multiple SNPs using MAS can be a viable option for improving rice varieties [69]. This variation in phenotypic traits among diverse germplasm and between the subpopulations is an indication that genetic variation is available in specific subpopulations and could be utilized in a wide diversity of rice-breeding programs. However, the IND subpopulation had more desirable salt tolerant traits (Table 1) and desirable alleles at the identified SNPs than AUS or TRJ (TEJ was only represented by two accessions, and hence was not analyzed independently). 

SNP 1_2594296 was identified with the alternate allele associated with a lower SSI score and increased leaf number and biomass in the IND group, although this allele is also found in moderate abundance in the TEJ and AUS subpopulations (Table 3a,b). The salt-tolerant alternate allele is predicted in 20.3% of IND suggesting this could be selected for improving salt tolerance when breeding within IND. It should be noted that in this study, the alternate allele does not appear to be associated with the phenotype in the AUS subpopulation, so for using this SNP in other subpopulations via introgression and MAS, it will be necessary to verify that the introgression is from an IND source that is likely to be linked with the functional variant for salt tolerance and not from an AUS source. SNP S1_33398135 has an alternate allele that is found almost exclusively in IND and is associated with an increased value for the Δ PHT trait under salt conditions. Likewise, SNP 2_2053382 should be useful in rice breeding because it has an allele that was found almost exclusively in IND and was associated with a reduced SSI score and increased Δ PHT, Δ green leaf number, and the biomass traits. 

A study by Shi et al. [70] identified a QTL for vigor index (a measure of seed germination and shoot elongation during salt stress) on rice chromosome 2 that occurred 1.38 Mb proximal to the SNP S2_2053382 region identified in our study. The Shi et al. study utilized 478 entries [305 *Indica*, and 85 *Japonica*, 65 *Aus*, 16 Basmati (ARO), and seven intermediate (admixture) accessions] with 6,361,920 SNPs from the 3000 genome data set and performed a GWAS for seed germination under 60 mM of salt (NaCl) stress. This locus may be another useful target for MAS to improve salt tolerance using diversity from IND. The Os02g0452 gene encodes a G-protein γ subunit and is reported to be a signal transducer during salt stress, and is a likely candidate for salt stress tolerance in this region [52,71]. 

SNP S3_17374343 is located within a broad GWAS peak spanning the centromere of chromosome 3 (18.9–19.9 Mb). TRJ and TEJ groups have almost exclusively the reference allele. Variability at the SNP is limited primarily to the AUS and IND groups where the alternate allele is associated with an increased SSI score, and reduced Δ green leaf number and biomass. Thus, this is an alternate allele to be selected against in rice breeding for improving salt tolerance. However, MAS may be complicated in this area due to the recombination suppression that occurs near the centromeric region, which also contains several important yield-related genes [72]. SNP S3_36149293 has an alternate allele found in the AUS group that is associated with a reduced SSI score and increased Δ green leaf number and biomass. This salt tolerance-associated allele from AUS may be a useful target for MAS to improve salt tolerance by introgression to the rice-breeding lines of other subpopulations. Gibberellin 20 oxidase, GA20OX1, is a likely candidate gene for this locus. GA20OX1 is a paralog of the semi-dwarf Sd-1 “green revolution” gene that has been found to increase cytokinin activity in rice [73]. The alternate allele of SNP S4_31361839 is the most frequent allele in all subpopulations except in TEJ, and is associated with an increased SSI score, as well as reduced Δ green leaf number and biomass. The reference allele is associated with higher salt tolerance and is found in most TEJ accessions (81.1%) and a moderate number of IND accessions (26.5%). Either TEJ or IND could be used as a source of salt tolerance alleles in MAS for this locus to improve the germplasm in other subpopulations. 

A GWAS study performed by Patishtan et al. [30] examined 306 rice accessions utilizing a 700 K high-density SNP chip after three durations of 50-mM salt treatments: short (6 h), medium (7 d), and long (30 d) on 15-day-old seedlings. Eight of their identified SNPs occurred within 1.5 Mb or less of the representative SNPs identified in this study. They reported two SNPs associated with d7 shoot potassium level on chromosome 1 and on chromosome 4, which are in proximity to the S1_2594296 and S4_31361839 SNPs of our study. Four SNPs on chromosome 10 associated with 6-h shoot sodium level were identified near SNP S10_11743519, which was identified in this study. A SNP each for the 6-h shoot sodium level and d30 shoot sodium level were identified on chromosome 10 near the SNP S10_18801757. GLP4-1, an auxin-binding gene, is a likely candidate gene for this locus [74]. The SNP 10_11743519 alternate allele was found primarily in the TEJ (41.9%) and TRJ (22.8%) groups, and was associated with a reduced SSI score, and increased Δ PHT, Δ green leaf number, and biomass. 

We identified two SNPs, S10_11743519 and SNP 10_18801757, on chromosome 10. Genomic regions in the proximity of these SNPs were also identified in a GWA study by Kumar et al. [75] that examined salt stress using a 6K SNP chip and 220 rice accessions. The authors identified a SNP within 1.3 MB of S10_11743519 that was associated with spikelet fertility and productive tillers under season-long EC 10 dS m^−1^ salt (7 NaCl: 1 Na_2_SO_4_: 2 CaCl_2_) stress in field conditions. The salt tolerance-associated alternate allele would be a good target for MAS to improve salt tolerance within TEJ or through the introgression of the TEJ or TRJ allele into IND or AUS. This locus is an example of the diversity from *Japonica* contributing to salt tolerance, whereas other reported loci derive tolerance from IND or AUS sources. ERF51, a likely candidate gene located in this region (Table 4), is also reported to be associated with cold tolerance [76]. The SNP 10_18801757 alternate allele is found primarily in AUS and IND subpopulations, and is associated with lower SSI scores, and increased Δ PHT, Δ green leaf number, and biomass. The salt tolerance-associated allele from IND or AUS would be a good target for MAS to improve salt tolerance within these groups or to introgress into other subpopulations such as TRJ or TEJ. As discussed above, six SNPs (1_2594296, 2_2053382, S3_36149293, S4_31361839, 10_11743519, and 10_18801757) in this study were identified as being useful for MAS.

In total, the study identified six potential novel loci that are promising targets for MAS with tolerant alleles coming from the *Indica* subspecies in five cases and the *Japonica* subspecies in one case. Pyramiding multiple salt tolerance alleles into a single genetic background is possible through molecular breeding, and may result in highly salt tolerant germplasm that could be a useful parent for rice-breeding programs. This study presents previously untapped genetic variation and the identification of novel rice germplasm that can be used to improve salt tolerance in rice, which in turn can help increase rice production to meet the demands of the world’s increasing population.

## 4. Materials and Methods 

### 4.1. Plant Material, Growth Conditions, Salt Stress Treatment

A group of 162 rice accessions (30 AUS, 93 IND, two TEJ, and 37 TRJ) representing 55 rice-growing countries was selected from the URMC along with 24 modern varieties from a Vietnamese breeding program and seven accessions with known salt tolerance or salt susceptibility along with three salt-tolerant checks (Pokkali, IR45427-2B-2-2B-1-1, and IR65196-3B-5-2-2) and three salt-sensitive checks (IR29, Nerica 6, and A69-1) were used to evaluate salt tolerance at the seedling stage (Table S3). Of these, DNA sequence data was available for 118 URMC accessions [30 AUS, 58 IND, and 30 *Japonicas* (two TEJ, and 28 TRJ)] [23,24] for genomic analysis. Hydroponic experiments were conducted under greenhouse conditions at the USDA-ARS Dale Bumpers National Rice Research Center, Stuttgart, Arkansas, USA. Seeds were planted according to the protocol of Gregorio et al. [68,77] with minor modifications. Briefly, each experimental unit was represented by two healthy seeds placed into each of 5 wells (1.5-cm diameter) that had been created in a 30 cm (L), 27 cm (W), 1.5 cm (D) Styrofoam floater with a fiberglass mesh attached to the bottom to support the rice seeds in the nutrient solution. Each floater was put in an individual 11.4-L black plastic tub that was 31.8 cm (L), 29 cm (W), 12.2 cm (D) in dimension. Twenty entries, including two repetitive check entries, Pokkali (resistant check) and IR 29 (susceptible check), were planted in each floater in a tub. The experiment was conducted as a randomized complete block design with three replications. Once seedlings were approximately 2 cm tall, the seedlings were thinned to one plant per well. When the seedlings were at the two-leaf stage, airsoft 6-mm pellets (Soft Air, Dallas, TX, USA) were added to each well to support the plant. Aluminum foil was wrapped around each tub and floater to prevent sunlight from entering the nutrient solution to help prevent algae formation. When >80% of seedlings were at the three-to-four leaf stage (generally about two weeks after germination), salt treatment was started (designated as day 0 or d_0) by adding NaCl (salt) to the nutrient solution. Electrical conductivity (EC) was kept constant at 6.0 ± 0.2. The pH (5 ± 0.2 and EC (6.0 ± 0.2) were adjusted every other day using 1 M of NaOH and 1 M of HCl. Nutrient solution was changed once a week to ensure the availability of sodium ions (Na^+^) for toxicity induction in rice seedlings. A side-by-side control treatment (without salt) was run on the check entries for quality control measures of the salt treatments. 

### 4.2. Phenotypic Data Collection, Processing, and Analysis

Phenotypic symptoms of sodium toxicity in the form of salt stress injury (SSI) scores were evaluated on the 10th day (d10) and 16th day (d16) after subjecting the seedlings to salt treatment. SSI scores were recorded on a scale of 1 (tolerant) to 9 (susceptible), as suggested by Gregorio et al. [77] and shown in Figure S3. In this investigation, we also measured the following seven early vigor traits that were related to plant growth and development [78]: plant height on the day of initiating salt stress (PHT_d0), plant height on day 10 of salt stress (PHT_d10), plant height on day 16 of salt stress (PHT_d16), green leaf number on the day of initiating salt stress (GLN_d0), green leaf number on day 14 of salt stress (GLN_d14), shoot biomass on day 16 of salt stress (SB_d16), and root biomass on day 16 of salt stress (RB_d16). On completion of the 16^th^ day of salt treatment, the rice seedlings were harvested by cutting them into two portions at the crown and dried individually at 60 °C for 96 h to determine the dry weights of: shoot biomass (SB) and root biomass (RB) separately.

The following four values for traits of interest were calculated as below:

Growth in plant height under 10 days of salt stress (Δ PHT-d10): PHT_d10 – PHT_d0
Growth in plant height under 16 days of salt stress (Δ PHT-d16): PHT_d16 – PHT_d0
Green leaf number (GLN) produced under 14 days salt stress (Δ green leaf#): GLN_d14 – GLN_d0
Total biomass after 16 days of salt stress (TB): SB_d16 + RB_d16

The best linear unbiased prediction (BLUP) estimates of the means for all traits were used for genomic analysis as explained by Eizenga et al. [79]. Briefly, to estimate the BLUP values for each measured and calculated trait, the variation among rice accessions was considered as a random effect. In the model, a normal distribution was used for all the studied traits. The values for each parameter were the means of three independent replications where each replication had five plants. An ANOVA was performed on growth variables using the standard least squares procedure of JMP13.2 (SAS Institute, Cary, NC, USA). Tukey’s HSD (Honestly Significant Difference) analysis was used for the comparisons of the growth parameters measured among the genotypes. Pearson’s correlation coefficients were determined between pairs of traits using JMP14.3.0 (SAS Institute, Cary, NC, USA).

### 4.3. Genome-Wide Association Mapping, Identification of Single-Nucleotide Polymorphisms (SNPs) and Candidate Genes

The USDA rice mini-core collection (URMC) was sequenced to an average read depth of 1.5x by Wang et al. [24], and the raw reads were later recalled as described in Huggins et al. [23]. A mixed linear model (MLM) in Tassel version 5 [80,81] was used to perform a genome-wide association study (GWAS) on 118 accessions in the URMC. Of these 118 accessions, six panels were used to capture the genetic diversity present that is specific to each subpopulation/subspecies. The first panel, “All”, was comprised of 118 accessions with 3,029,968 SNPs and contained four rice subpopulations present in this study; IND, AUS, TEJ, and TRJ. No aromatic accessions from the URMC were used in this study. The “IND” panel contained 58 *indica* subpopulation accessions and 1,883,603 SNPs. The “AUS” panel had 30 *aus* accessions and 1,753,773 SNPs. The “TRJ” panel had 28 *tropical japonica* accessions and 1,067,722 SNPs. Only two *temperate japonica* accessions represented the *Japonica* subspecies.

For each panel, SNPs were filtered to a minimum allele frequency of 0.05 to remove rare alleles, and SNPs with 20% or greater missing calls were removed. Tassel version 5 [80] was used to generate a centered identity by state kinship matrix and principal component analysis (PCA) for each filtered panel. The kinship matrix and first three principal components were used in the MLM and the options of no compression of the kinship matrix and the variance components estimated for each marker-trait combination were used. 

The R script qqman [82] was used to generate Manhattan and Q-Q plots. For exploratory purposes, SNPs with −log10 of p values >5 were considered significant, and increased stringency was applied during data interpretation as needed. A Perl script was used to define the associated chromosome regions from individual SNPs or groups of physically linked SNPs. Chromosome regions encompassed 50 Kb in both directions around each individual significant SNP, and were extended to include nearby significant SNPs occurring within 200 Kb. The Perl script designated a ‘Peak SNP’ for each region, which corresponded to the SNP with the most significant p-value found within the region. 

A Perl script was used to help identify candidate gene(s) within 250 Kb of the significant regions, as described in Huggins et al. [23]. This Perl script used gene annotation from MSU7 [83] and the Rice Annotation Project (RAP1; http://rapdb.dna.affrc.go.jp/, accessed 14 September 2018) [84] plus two new annotation files that were created to aid in candidate gene identification. The first file was based on the results of the candidate genes identified in Cohen and Leach [22], which examined general biotic and abiotic stress response genes in rice. The second file merged all MSU7 annotations [83] (accessed 30 May 2019) with data in Oryzabase (OrzbaseGeneListEn_20190424010057; https://shigen.nig.ac.jp/rice/oryzabase/download/gene, accessed 24 April 2019) that shared an RAP ID. As described in Huggins et al. [23], an additional Perl script was used to examine the regions that overlapped across the different traits and subpopulation/subspecies panels. 

SNPs were grouped into chromosomal regions by processing GWAS results with custom Perl scripts using the methods reported by Huggins et al. [23]. Based on p-values and allele effects, chromosomal regions and corresponding peak SNPs that are highly predictive of SSI scores and related phenotypes were selected for detailed analysis. Allele effects, significance, and R-squared values for each of the selected SNPs were calculated separately for each subpopulation by ANOVA in JMP 14.0.0. To determine allele distribution among a larger panel of rice lines than the URMC alone, we further examined the representative significant SNPs in public databases. The allele frequency information was computed by using RiceVarMap, which contains genotype information on 4726 rice accessions [49]. If allele frequency information was not present in RiceVarMap, then it was computed from the SNP-seek database, which contains genotype information on 3000 rice accessions [48,85].

## Figures and Tables

**Table:** (**a**)

Classification	Genotype	SSI Score-d10	SSI Score-d16	Δ PHT-d10 (cm)	Δ PHT-d16 (cm)	Δ Green Leaf#-d14	Total Biomass Plant^−1^ (g)	Shoot Biomass Plant^−1^ (g)	Root Biomass Plant^−1^ (g)
I	Sensitive checks	5.45 ^a^	7.53 ^a^	8.25 ^c^	8.29 ^d^	−2.97 ^d^	0.16 ^d^	0.13 ^d^	0.03 ^d^
	Tolerant checks	1.38 ^d^	2.32 ^d^	22.44 ^a^	29.43 ^a^	0.64 ^a^	1.30 ^a^	1.08 ^a^	0.22 ^a^
	URMC *	4.61 ^b^	6.39 ^b^	12.98 ^b^	14.07 ^b^	−2.06 ^c^	0.28 ^c^	0.23 ^c^	0.05 ^c^
	Vietnamese lines *	3.52 ^c^	4.94 ^c^	10.45 ^c^	12.06 ^c^	−1.24 ^b^	0.35 ^b^	0.29 ^b^	0.07 ^b^
II *	AUS	4.46 ^B^	6.50 ^B^	12.96 ^A^	13.94 ^A^	−2.08 ^A,B^	0.27 ^B^	0.22 ^B^	0.05 ^B^
	IND	3.95 ^C^	5.48 ^C^	12.61 ^A^	14.00 ^A^	−1.76 ^A^	0.34 ^A^	0.27 ^A^	0.07 ^A^
	TEJ	3.20 ^C^	4.93 ^B,C^	14.32 ^A^	15.45 ^A^	−1.33 ^A,B^	0.44 ^A^	0.36 ^A^	0.08 ^A^
	TRJ	5.63 ^A^	7.55 ^A^	12.04 ^A^	12.89 ^A^	−2.24 ^B^	0.20 ^C^	0.16 ^C^	0.04 ^C^
	ALL ^δ^	4.43 ± 0.07	6.15 ± 0.09	12.57 ± 0.20	13.75 ± 0.23	−1.93 ± 0.06	0.29 ± 0.01	0.24 ± 0.01	0.06 ± 0.001

* Checks (tolerant and sensitive) were excluded from calculations for the LSMeans and Tukey’s HSD analysis. Column values not followed by the same letter within a classification are significantly different (*α* = 0.050). Note: Lowercase letters are used for comparisons in I, and uppercase letters are used for comparisons in II. ^δ^ ALL in this table denotes all 162 entries.

**Table:** (**b**)

Genotype	Min/Max	SSI Score-d10	SSI Score-d16	Δ PHT-d10 (cm)	Δ PHT-d16 (cm)	Δ Green Leaf#-d14	Total Biomass Plant^−1^ (g)	Shoot Biomass Plant^−1^ (g)	Root Biomass Plant^−1^ (g)
Sensitive check	Min	3.80	5.20	2.60	2.60	−4.00	0.11	0.01	0.01
	Max	7.40	9.00	11.30	11.30	−2.00	0.27	0.21	0.06
Tolerant check	Min	1.00	1.00	6.00	8.80	−1.00	0.42	0.35	0.07
	Max	3.80	4.60	30.20	47.70	2.00	1.99	1.66	0.33
URMC	Min	1.00	1.80	3.60	4.00	−4.00	0.03	0.03	0.01
	Max	9.00	9.00	27.70	30.40	1.00	0.99	0.81	0.18
Vietnamese lines	Min	1.40	2.00	0.00	4.40	−5.00	0.14	0.11	0.02
	Max	6.60	8.20	18.50	20.70	1.00	0.81	0.67	0.15
AUS *	Min	1.30	2.00	5.00	5.00	−4.00	0.09	0.07	0.02
	Max	7.40	9.00	20.50	25.90	1.00	0.81	0.66	0.14
IND *	Min	1.00	1.80	0.00	4.00	−5.00	0.12	0.09	0.02
	Max	7.40	9.00	27.70	30.40	1.00	0.99	0.81	0.18
TEJ *	Min	1.60	2.00	11.50	11.50	−3.00	0.21	0.17	0.04
	Max	4.60	7.20	17.10	19.10	1.00	0.71	0.58	0.13
TRJ *	Min	3.00	3.20	4.70	4.70	−4.00	0.03	0.03	0.01
	Max	9.00	9.00	25.50	30.40	0.00	0.48	0.39	0.09
ALL *	Min	1.00	1.80	0.00	4.00	−5.00	0.03	0.03	0.01
	Max	9.00	9.00	27.70	30.40	1.00	0.99	0.81	0.18

* Checks (tolerant and sensitive) were excluded from calculations for these values.

**Table 2:** Correlation coefficients among seedling salt tolerance traits determined under hydroponic conditions (N = 162).

	SSI Score-d10	SSI Score-d16	Δ PHT-d10	Δ PHT-d16	Δ Green Leaf#-d14	Total Biomass	Shoot Biomass	Root Biomass
SSI score-d10	1							
SSI score-d16	0.9041 *	1						
Δ PHT-d10	−0.4270 *	−0.4309 *	1					
Δ PHT-d16	−0.5366 *	−0.5708 *	0.9367 *	1				
Δ green leaf#-d14	−0.7985 *	−0.8577 *	0.4030 *	0.5393 *	1			
Total biomass	−0.7218 *	−0.7495 *	0.5838 *	0.7177 *	0.7170 *	1		
Shoot biomass	−0.7177 *	−0.7417 *	0.5885 *	0.7191 *	0.7112 *	0.9991 *	1	
Root biomass	−0.7267 *	−0.7696 *	0.5447 *	0.6918 *	0.7286 *	0.9809 *	0.9722 *	1

* = *p* < 0.0001.

**Table:** (**a**)

SNP ID	Chr.	Position (bp)	Ref. Allele	Alt. Allele	Sub-Pop with Alt. Allele Associated with Traits	Traits Associated with the Alt. Allele	Frequencies for the Ref. Allele				
							TRJ	TEJ	ARO	AUS	IND
**S1_2594296**	1	2,594,296	G	C	IND	SSI score, Δ green leaf#, biomass	95.7%	50.2%	85.9%	64.1%	79.7%
**S1_33398135**	1	33,398,135	C	T	IND	Δ PHT	100.0%	99.9%	100.0%	98.9%	43.2%
**S2_2053382**	2	2,053,382	A	C	IND	SSI score, Δ PHT, Δ green leaf#, biomass	99.9%	100.0%	100.0%	99.2%	65.7%
**S3_17374343**	3	17,374,343	C	T	AUS, IND	SSI score, Δ green leaf#, biomass	97.2%	99.7%	19.8%	91.4%	10.3%
**S3_21582523**	3	21,582,523	C	T	AUS, IND	SSI score, Δ green leaf#, biomass	90.1%	98.0%	5.2%	41.3%	44.2%
**S3_36149293**	3	36,149,293	A	T	AUS	SSI score, Δ green leaf#, biomass	99.8%	100.0%	100.0%	57.2%	98.4%
**S4_31361839**	4	31,361,839	G	A	IND	SSI score, Δ green leaf#, biomass	2.6%	81.1%	1.0%	0.7%	26.5%
**S10_11743519**	10	11,743,519	C	T	TRJ	SSI score, Δ PHT, Δ green leaf#, biomass	77.2%	58.1%	100.0%	100.0%	99.6%
**S10_18801757**	10	18,801,757	A	C	AUS, IND	SSI score, Δ PHT, Δ green leaf#, biomass	96.6%	50.2%	95.8%	63.9%	86.1%

* All the frequencies for the reference alleles were computed using the SNP-seek database of the 3000 rice genomes [48] and RiceVarMap [49]. TRJ = *Tropical japonica;* TEJ = *Temperate japonica*; *ARO = aromatic*; AUS = *Aus*; IND = *Indica*. Chr. denotes chromosome number.

**Table:** (**b**)

SNP	Sub-Pop	Traits	Prob>|t|	Rsquare	Alternate Allele Effect
1_2594296	IND	d10 SSI score	0.0007	0.32	−1.247
		d16 SSI score	<0.0001	0.47	−2.152
		Δ green leaf#-d14	<0.0001	0.43	1.043
		Shoot biomass d16	<0.0001	0.45	0.126
		Root biomass d16	<0.0001	0.65	0.035
1_33398135	IND	Δ PHT-d10	<0.0001	0.48	3.543
		Δ PHT-d16	<0.0001	0.44	3.998
2_2053382	IND	d10 SSI score	0.0020	0.22	−0.904
		d16 SSI score	0.0002	0.30	−1.659
		Δ PHT-d10	0.0935	0.07	1.536
		Δ PHT-d16	0.0426	0.10	2.215
		Δ green leaf#-d14	0.0002	0.30	0.822
		Shoot biomass d16	<0.0001	0.39	0.118
		Root biomass d16	<0.0001	0.53	0.036
3_17374343	AUS	d10 SSI score	0.0110	0.28	1.200
		d16 SSI score	0.0974	0.13	1.024
		Shoot biomass d16	0.0196	0.24	−0.079
		Root biomass d16	0.0232	0.23	−0.017
	IND	d10 SSI score	<0.0001	0.31	1.708
		d16 SSI score	<0.0001	0.32	2.422
		Δ green leaf#-d14	0.0001	0.29	−1.117
		Shoot biomass d16	0.0014	0.21	−0.123
		Root biomass d16	<0.0001	0.31	−0.038
3_21582523	AUS	d10 SSI score	0.0008	0.51	1.336
		d16 SSI score	0.0052	0.40	1.509
		Δ green leaf#-d14	0.0200	0.29	−0.649
		Shoot biomass d16	0.0116	0.34	−0.079
		Root biomass d16	0.0042	0.41	−0.018
	IND	d10 SSI score	0.0010	0.33	0.987
		d16 SSI score	0.0006	0.36	1.460
		Δ green leaf#-d14	0.0006	0.36	−0.740
		Shoot biomass d16	0.0231	0.18	−0.069
		Root biomass d16	0.0135	0.21	−0.018
3_36149293	AUS	d10 SSI score	0.0124	0.25	−0.800
		d16 SSI score	0.0008	0.41	−1.426
		Δ green leaf#-d14	0.0011	0.39	0.686
		Shoot biomass d16	0.0331	0.19	0.051
		Root biomass d16	0.0089	0.27	0.013
4_31361839	IND	d10 SSI score	0.0196	0.40	1.113
		d16 SSI score	0.0151	0.43	1.654
		Δ green leaf#-d14	0.0171	0.42	−0.816
		Shoot biomass d16	0.0003	0.71	−0.164
		Root biomass d16	0.0004	0.69	−0.040
10_11743519	TRJ	d10 SSI score	<0.0001	0.58	−1.248
		d16 SSI score	<0.0001	0.66	−1.603
		Δ PHT-d10	0.0416	0.21	2.016
		Δ PHT-d16	0.0086	0.33	3.237
		Δ green leaf#-d14	0.0004	0.51	0.710
		Shoot biomass d16	0.0007	0.48	0.077
		Root biomass d16	0.0033	0.39	0.015
10_18801757	AUS	d10 SSI score	0.0001	0.46	−1.185
		d16 SSI score	<0.0001	0.53	−1.647
		Δ green leaf#-d14	<0.0001	0.46	0.819
		Shoot biomass d16	0.0103	0.24	0.048
		Root biomass d16	0.0028	0.30	0.013
	IND	d10 SSI score	0.0122	0.13	−0.817
		d16 SSI score	0.0007	0.22	−1.579
		Δ PHT-d16	0.0918	0.06	1.948
		Δ green leaf#-d14	0.0013	0.20	0.723
		Shoot biomass d16	0.0006	0.23	0.100
		Root biomass d16	0.0005	0.23	0.026

**Table 4:** Candidate genes in the chromosomal region of representative SNPs associated with salt stress tolerance at the seedling stage in rice.

SNP	MSU7 Locus ID	Position	Gene Name	Putative Function	Reference
S1_2594296	LOC_Os01g04800	Chr01:2202264 - 2203860	AP2/EREBP129	Transcriptional reprogramming during salt stress	[50]
S1_33398135	LOC_Os01g57610	Chr01:33308448 - 33311391	OsGH3.1	Auxin-responsive role in salinity tolerance	[51]
S2_2053382	LOC_Os02g04520	Chr02:2007232 - 2009961	G-protein γ subunit	Signal transducer during salt stress	[52]
	LOC_Os02g04630	Chr02:2070492 - 2075369	Vacuolar cation/proton exchanger	Protects primary cell mechanisms mediated by ion homeostasis	[53]
S3_17374343 (and S3_21582523)	LOC_Os03g30420	Chr03:17340415 - 17340601	GL3.2	Cytochrome P450, stress tolerance	[22,54]
	LOC_Os03g31550	Chr03:17985563 - 17998498	Aldehyde oxidase putative	Abiotic stress response	[22,55]
	LOC_Os03g31750	Chr03:18153143 - 18160127	Pyruvate orthophosphate dikinase (PPDK)	Improved plant physiology under abiotic stress	[56]
	LOC_Os03g32314	Chr03:18485606 - 18488371	AOC1, allene oxide cyclase	Response to salt and other abiotic stresses	[57]
	LOC_Os03g33590	Chr03:19197601 - 19204090	Interferon-related developmental regulator	Salt sensitivity in Arabidopsis	[22]
	LOC_Os03g36730	Chr03:20363507 - 20370047	OST3/OST6 family protein	Hypersensitivity to osmotic and salt stress	[58]
	LOC_Os03g37260	Chr03:20650253 - 20653294	Pentatricopeptide repeat	Post-transcriptional gene regulation under abiotic stresses	[59]
S3_36149293	LOC_Os03g63970	Chr03:36152044 - 36152517	GA20OX1, gibberellin 20 oxidase 1	Growth and development, downregulated under abiotic stress	[22]
S4_31361839	LOC_Os04g52720	Chr04:31392876 - 31395185	GLP4-1, germin-like protein 4-1	Salt and osmotic stress tolerance	[60]
S10_11743519	LOC_Os10g22600	Chr10:11731592 - 11732917	ERF51, ethylene response factor 51	Osmotic stress tolerance	[61]
S10_18801757	LOC_Os10g35150	Chr10:18754181 - 18758096	Expressed protein	Downregulated during abiotic stress	[22]
	LOC_Os10g35460	Chr10:18976009 - 18979110	Phytochelatin synthase	Regulation of osmolytes, Na^+^/K^+^ ratio, sequestration in the vacuole for cellular redox homeostasis	[22,62]

## Supplementary Materials

The following are available online at https://www.mdpi.com/2223-7747/8/11/472/s1. Figure S1: Manhattan and quantile–quantile (Q-Q) plots for the entire GWA analysis conducted in this study, Figure S2: Violin plots showing effect of each identified significant SNP/allele (x axis) on the salt stress injury (SSI) score on d16 (y axis) of salt stress in each rice subpopulations (x axis), Figure S3: Representative pictures of criteria used for salt stress injury (SSI) scoring for salt tolerance, Table S1: Significant regions identified across all subpopulations/panels for all measured traits, Table S2: Overlapping significant regions identified across all subpopulations/panels for all measured traits, Table S3: A complete list of the rice accessions evaluated for salt tolerance at three-four leaf seedling stage.
